# Prefabrication of 3D Cartilage Contructs: Towards a Tissue Engineered Auricle – A Model Tested in Rabbits

**DOI:** 10.1371/journal.pone.0071667

**Published:** 2013-08-09

**Authors:** Achim von Bomhard, Johannes Veit, Christian Bermueller, Nicole Rotter, Rainer Staudenmaier, Katharina Storck, Hoang Nguyen The

**Affiliations:** 1 Department of Maxillofacial Surgery, Technical University of Munich, Munich, Germany; 2 Department of Otorhinolaryngology, Ulm University Medical Center, Ulm, Germany; 3 HNO Praxis im Zentrum, ENT and Plastic Surgery, Munich, Germany; 4 Department of Otorhinolaryngology, Technical University of Munich, Munich, Germany; 5 Department of Hand- and Microsurgery, Military Hospital 108, Hanoi, Vietnam; University of Minho, Portugal

## Abstract

The reconstruction of an auricle for congenital deformity or following trauma remains one of the greatest challenges in reconstructive surgery. Tissue-engineered (TE) three-dimensional (3D) cartilage constructs have proven to be a promising option, but problems remain with regard to cell vitality in large cell constructs. The supply of nutrients and oxygen is limited because cultured cartilage is not vascular integrated due to missing perichondrium. The consequence is necrosis and thus a loss of form stability. The micro-surgical implantation of an arteriovenous loop represents a reliable technology for neovascularization, and thus vascular integration, of three-dimensional (3D) cultivated cell constructs. Auricular cartilage biopsies were obtained from 15 rabbits and seeded in 3D scaffolds made from polycaprolactone-based polyurethane in the shape and size of a human auricle. These cartilage cell constructs were implanted subcutaneously into a skin flap (15×8 cm) and neovascularized by means of vascular loops implanted micro-surgically. They were then totally enhanced as 3D tissue and freely re-implanted in-situ through microsurgery. Neovascularization in the prefabricated flap and cultured cartilage construct was analyzed by microangiography. After explantation, the specimens were examined by histological and immunohistochemical methods. Cultivated 3D cartilage cell constructs with implanted vascular pedicle promoted the formation of engineered cartilaginous tissue within the scaffold in vivo. The auricles contained cartilage-specific extracellular matrix (ECM) components, such as GAGs and collagen even in the center oft the constructs. In contrast, in cultivated 3D cartilage cell constructs without vascular pedicle, ECM distribution was only detectable on the surface compared to constructs with vascular pedicle. We demonstrated, that the 3D flaps could be freely transplanted. On a microangiographic level it was evident that all the skin flaps and the implanted cultivated constructs were well neovascularized. The presented method is suggested as a promising alternative towards clinical application of engineered cartilaginous tissue for plastic and reconstructive surgery.

## Introduction

Tissue engineering is a promising method for repair or replacement of any tissues in the human body that is injured or damaged as a result of disease or trauma. The first cultivated cartilage cell constructs have already been described in the early 70′s by W.T. Green [1]. Nowadays, after 40 years of research, there continue to be problems in this field with regard to cell expansion, requirements regarding cell vitality in large cell constructs and homogenous seeding in cell carriers [2]. Therefore optimal cell carriers must be identified and implemented. Features with regard to biocompatibility, degradation, elasticity and mechanical stability, sufficient diffusion of nutrients into the center of the scaffold and suitable qualities for cell adhesion, growth, differentiation and proliferation play a decisive role.

Because cartilage is an avascular tissue consisting of chondrocytes only, which are embedded in a matrix composed of collagen and proteoglycans, the survival and growth of transferred native cartilage grafts depends in part on the maintenance of well vascularized perichondrium [3]. Engineered cartilage constructs with a large volume of expended cells usually require a hyperoxic environment for their growth and proliferation, and have no perichondrium [4]. So their blood and nutrient supply is clearly limited. This results in cell death and unavoidable necrosis of the engineered construct with subsequent loss of shape and function [2], [5].

With respect to clinical applications, we hypothesized that the survival and transplantation of tissue-engineered constructs should be more effective if they, or their immediate surrounding, are neovascularized and transferred as an axial free flap based on a reliable vascular pedicle. Our previous experimental studies in chinchilla-bastard rabbits have revealed the reliable ability of neovacularization and free microsurgical transplantation of small engineered cartilage constructs using prefabricated flaps [6]. The present study was designed to investigate the ability of combining flap prefabrication and large engineered constructs with free microsurgical transplantation to improve cell survival especially in the center of the engineered cartilage constructs. Prefabrication is a clinically established approach from reconstructive surgery for the neovascularization of skin flaps. The principle of prefabricated flaps is based on the transformation of a previously non-axial perfused tissue area into an axially supplied flap by means of the implantation of an arteriovenous vascular pedicle [7]. From the implanted vascular pedicle, new vessels sprout into the skin flaps and connect with the originally existing vascular system. The new axially supplied skin flap formed in this way can – based on the implanted vascular pedicle – be well neovascularised and can then be freely transplanted with a secure blood supply [8].

Neovascularization of native cartilage was first described by Hirase et. al. in 1988 [3]. They dissected the central vessels out of the pinna in rabbits, and implanted them in a subcutaneous (extraperichondrial) pocket of a random pattern, distally based, full-thickness ear flap. After three weeks the neovascularized chondrocutaneous flaps (2×3 cm) could be freely transferred to the contralateral pinna. This procedure was performed to prevent resorption, which is present in free cartilage grafts. Morrison et. al. implanted the femoral artery and vein of rabbits in various vascular configurations, directly into the subdermal layer of the skin, in an attempt to fabricate a thin axial-pattern flap, containing only skin and immediately underlying connective tissue, which is significantly thinner than any free flap previously available [9]. In 1993, Walton et. al. demonstrated the feasibility to engineer a fibrovascular stroma to a biomaterial (expanded polytetrauoroethylene 2×2×0.2 cm) using an isolated vascular pedicle and produce a synthetic ap capable of supporting an overlying skin graft [10]. Kataras et al. prefabricated composite arteriovenous aps with implantation of an autologous graft (cartilage) or an alloplastic material (porous polyethylene) in rabbits, and indicated that arteriovenous perfusion can nourish a prefabricated ap containing an implanted material [11].

Unlike other authors, we have developed a new model of large tissue-engineered cartilage in shape and size of a human auricle, associated with large prefabricated aps (dimension of 8×15 cm) in chinchilla-bastard rabbits, based on the anatomic similarities between humans and rabbits. In order to design the study to be as close to clinical application as possible, we decided to adopt an autologous animal model and transplanted the cultivated 3D cartilage tissue in the form of a human auricle within a closed system. This had the advantage that no immunosuppression was required.

## Materials and Methods

All reagents, cell culture media and supplements were purchased from Sigma Aldrich (Munich, Germany), Invitrogen (Darmstadt, Germany) and Biochrom (Berlin, Germany) unless indicated otherwise.

The trials were conducted on female chinchilla-bastard rabbits (Charles River Laboratories, Sulzfeld, Germany). This study was carried out in strict accordance with European guidelines for the Care and Use of Laboratory Animals. The protocol was approved by the Committee on the Ethics of Animal Experiments of the Technical University of Munich (Permit Number: Reg. Obb. AZ 55.2-1-54-2532-20-07). All surgical procedures were performed under general anesthesia with infusion of ketamine 40 mg/kg and xylazine 4 mg/kg i.v., and all efforts were made to minimize suffering. All operations were carried out under sterile conditions. A total of five operations were carried out on each animal (only step (1) and (2) in the cartilage construct control group):

1. Auricular cartilage biopsy harvesting for the in vitro production of cultivated cartilage constructs using porous constructs made of polycaprolactone in the form of an auricle.
2. In vivo construct implantation in an abdominal flap measuring 8×15 cm, three to four weeks after cartilage biopsy.
3. In vivo neovascularization by means of the implantation of an arteriovenous vascular pedicle, one week after construct implantation.
4. 6 weeks after implantation of the vascular pedicle, the abdominal skin flap and immediately underlying connective tissue, 8×15 cm in size, centered over the course of the transposed vessels, was raised as a rectangular island flap to proof the ability of the neovascularized cultivated 3D cartilage cell construct to survive solely perfused by the newly implanted axial vascular pedicle. After elevation the flap was resutured into position and observed daily for circulatory viability and the presence of necrosis.
5. After wound healing the flap was raised again, with disconnection of the vascular pedivle, and was reanastomosed microsurgically to A. saphena and V. saphena magna to proof the possibility of free transplantation.

Total experimental time was 9 to 11 months.

### Harvesting of Cartilage, Cell Culture, and Seeding of Biomaterials

In the first operation an auricular cartilage biopsy 5×30 mm in size from the auricle of each rabbit was harvested in order to obtain autologous chondrocytes. The auricular cartilage biopsy was cleaned and minced into 1×1 mm pieces under sterile conditions. Chondrocytes were enzymatically isolated during 16 hours incubation at 37°C in collagenase II (1,108 U/ml) and resuspended with Dulbecco Modified Eagle medium, foetal calf serum 100 ml/L and Antibiotic/Antimycotic 24 ml/L. For amplification chondrocytes were cultured at 37°C and 5.5% CO_2_. Following the attachment of the cells, culture medium was changed twice to three times per week.

At subconfluence cells were removed after being washed in Phosphate Buffered Saline with trypsin 0.05% and then re-seeded at a concentration of 30,000 cells/cm^2^. After about two weeks, and, on average, two to three passages, it was possible to achieve a sufficient cell count of approx. 20×10^6^ cells/cm^3^.

#### Biomaterial constructs

Polycaprolactone-based polyurethane scaffolds in the shape of human auricles were kindly manufactured by PolyMaterials, Kaufbeuren, Germany, as previously described [12].

**Figure 1:**
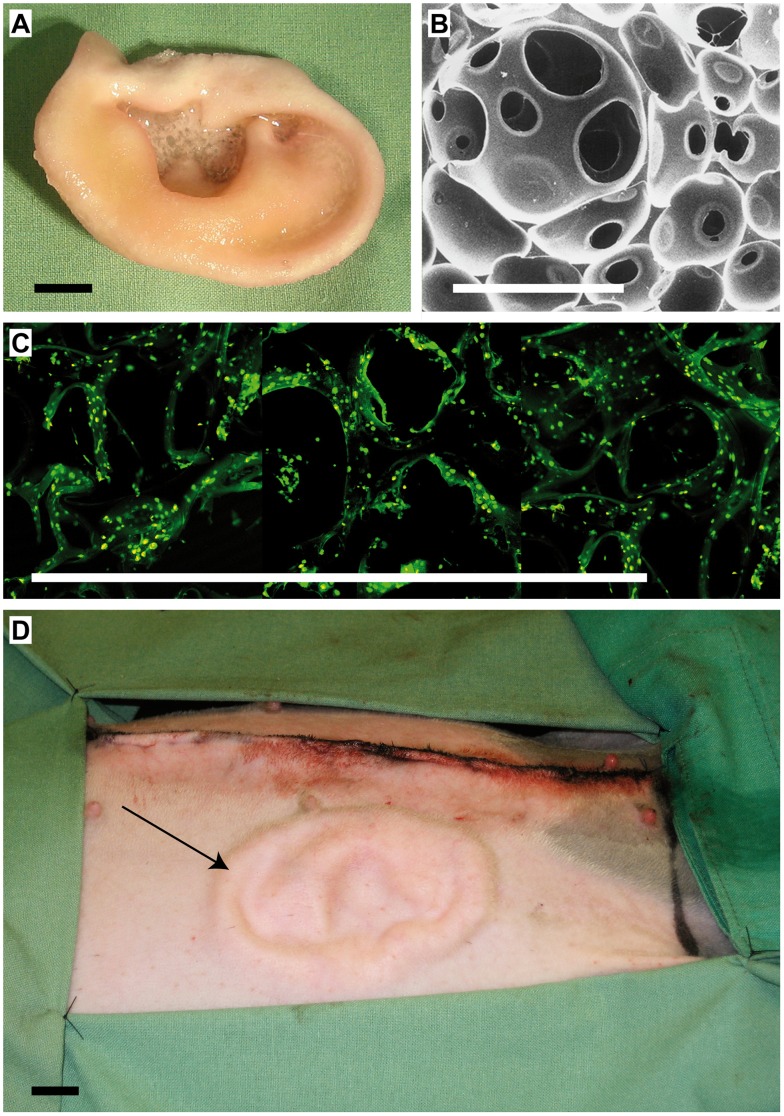
Biomaterial construct prior to implantation. A: Polyurethane based polycaprolactone construct seeded with chondrocytes. B: SEM picture of porous PCL construct, magnification 48×. C: Live/dead staining was performed with fluoresceindiacetate and propidium jodid using confocal laser scanning microscopy. D: Cultivated cartilage cell biomaterial construct (black arrow) implanted subcutaneously above the panniculus carnosus; day 0 postoperative. Scale bars = 1 cm.

Poly(caprolactone)diol (PCL diol) (M_n_ = 1250), poly(caprolactone)triol (PCL triol) (M_n_ = 900), DABCO DC-3042 (Air Products, Hattingen, Germany), dextrose, methylal, and diazabicycloundecene (Acros St. Augustin, Germany) were premixed and poured into one chamber of a two-chamber syringe equipped with a static mixer. The other chamber was filled with isophorone diisocyanate and methylal, and the syringe was heated to 37 to 38°C. The mixture was slowly injected into a silicone mold in the shape and size of a large (4×6 cm, 13 samples) and a small (3×4 cm, 2 samples) human auricle - created through rapid prototyping directly from computer aided design (CAD) data sources, via an STL file - and heated to 67 to 68°C for 2 h. Afterward, constructs were washed in boiling distilled water for 1 h and rinsed extensively with PBS, and autoclaved in a ask containing excess of PBS. The scaffold consisted mainly of PCL diol and PCL triol (39% each) as well as isophorone diisocyanate. The theoretical cross-linking percentage of the repeating units of polyol was 3%. Pore size was 180–250 µm with a porosity of ca. 85% *(* *fig. 1B* *)*. Characterization of the material in detail was published by Eyrich et. al. in 2007 [13].

#### Cell seeding

Biomaterial constructs were autoclaved in a flask containing excess of PBS. In order to orientate the polar segments of the polymer to the surface of the scaffold to improve cell adhesion ability of the biomaterial constructs and to disinfect, they were placed in EtOH 70% prior to cell seeding and then washed in Phosphate Buffered Saline. The cultivated cells were dissolved in thrombin (Baxter, Unterschleißheim, Germany) and resuspended in a thrombin buffer (5 U/ml pharmacy, University Hospital Rechts der Isar, Munich, Germany). The thrombin quantities constituted half of the absorption volumes of the biomaterial construct. In addition, the same quantity of fibrinogen – dissolved in Trasylol 100 mg/ml (Bayer, Leverkusen, Germany) was added to the mixture. The resulting cell-glue was, with the help of a syringe, (Becton Dickson, Heidelberg, Germany) and a cannula (B. Braun, Melsungen, Germany) injected into the biomaterial construct (30×10^6^ cells/cm^3^). The seeded construct *(* *fig. 1A* *)* was left in an incubator until it hardened (approx. 30 mins) and then covered in a cell culture vessel with DMEM culture medium. Foetal calf serum (100 ml/L), antibiotic/antimycotic (24 ml/L), insulin (2 µl/ml), proline (4 µl/ml) and vitamin C (5 µl/ml) were added and pre-cultivated in the incubator for 6 days prior to implantation. The medium was changed every other day. Before implantation, the biomaterial constructs were rinsed using an isotonic NaCl solution (B. Braun, Melsungen, Germany) to remove medium residues.

#### Neovascularization and free transplantation of cultivated cartilage cell constructs

Chondrocyte biomaterial cell constructs were first neovascularized in skin flaps by means of vascular pedicle implantation and then, based on the implanted axial vascular pedicle, were freely transplanted microsurgically back into position.

### Implantation of Chondrocyte Biomaterial Constructs

In the second operation implantation was performed on the right side of the abdomen, in a skin flap measuring 150×80 mm *(* *fig. 1D* *)*. Working on the basis of a medial craniocaudal incision of 150 mm, a pocket of approx. 80 mm in depth was prepared above the panniculus carnosus where the seeded construct was attached using 3–4 stitches Vicryl 4/0. In order to provide protection against manipulation of the rabbits, the wound suture for was carried out intracutaneously.

### Prefabrication of 3D Flaps by Vascular Pedicle Implantation

**Figure 2:**
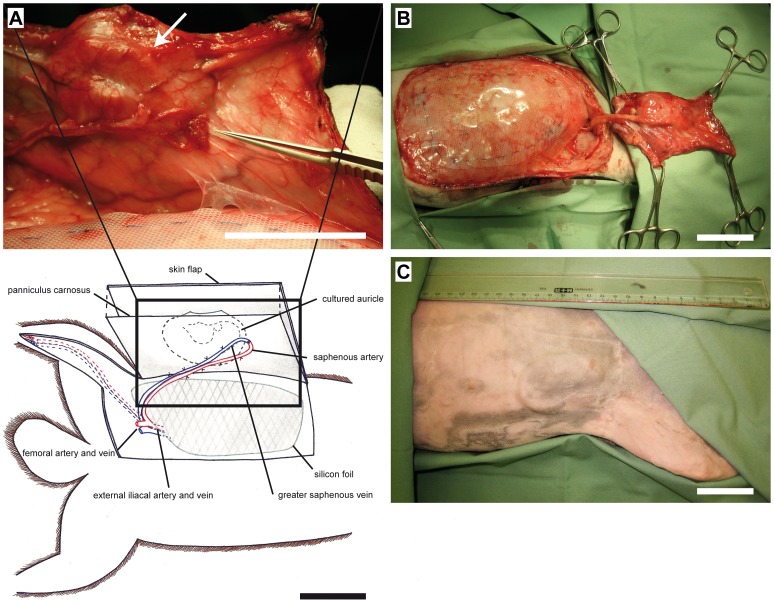
Surgical procedures. A: The implantation of the cultured auricle (white arrow) took place on the right side of the abdomen above the panniculus carnosus in a skin flap measuring 150×80 mm. A silicon foil was sewn onto the abdominal fascia to prevent neovascularization from the abdominal wall. Saphenous artery and greater saphenous vein were distally anastomosed turned in the cranial direction and attached below the abdominal skin flap. *indicates the original position of A. saphena and V. saphena magna. B: Four-sided flap excision. To proof the ability of the neovascularized cultivated 3D cartilage cell construct (black arrow) to survive solely perfused by the newly implanted axial vascular pedicle the abdominal flap was excised four-sided, leaving the vascular pedicle (white arrow) intact. C: Macroscopic view after free microsurgical transplantation. Healed skin flap three weeks after free microsurgical transplantation. Black arrow indicates the integrated neovascularized cultured auricle. Scale bars = 5 cm.

After wound healing in a third operation the planned skin flaps with implanted cartilage constructs were lifted laterally. Then, using ethilon 3/0, a silicon foil (LPI, Perouse, France, Bornel) with the dimensions 80×150×0.25 mm was sewn onto the abdominal fascia to prevent neovascularization from the abdominal wall. By means of an incision from the inguinal ligament to the calcaneus, saphenous artery and greater saphenous vein were exposed. The distal ends were anastomosed using 10/0 ethilon to achieve maximum blood flow through the newly created arteriovenous shunt [4]. The thus formed vascular pedicle was turned cranially and attached below the abdominal skin flap using Vicryl 8/0. The distance required to move the vascular pedicle was approximately 15 cm. *Figure 2A* gives an anatomical overview.

### Four-sided Flap Excision (Rectangular Island Flap)

After a neovascularization period of 21 days, prior to free transplantation, incisions were made around the flap in the same dimension, while keeping the newly implanted vascular pedicle intact *(* *fig. 2B* *)*, and were then sewn back in place to analyse the ability of the neovascularized 3D chondrocyte construct to survive solely by perfusion of the newly implanted axial vascular pedicle.

### Free Microsurgical Transplantation

After wound healing, the entire abdominal flap, with the integrated, cultivated auricular cartilage tissue was detached completely, vessels were disconnected, and then re-anastomosed in the same place using Prolene 10–0. The distal and proximal perivascular tissue was anastomosed using Prolene 7–0, in order to achieve strain relief for the anastomosis at the vascular pedicle. This prevented tearing of the vascular pedicle caused by movements of the rabbits.

### Control Groups

In the two control groups, a total of 8 animals were operated on. All incisions were hereby carried out in the same manner as in the test group. In the first control group (cartilage construct control group) autologous cartilage constructs as in the study group were implanted subcutaneously in the same position in two rabbits without the implantation of a vascular pedicle. In the second control group (microangiographic control) 6 animals served to determine the existing pattern of vessels and the morphology in the native abdominal skin flap.

### Evaluation Parameters

Flaps and construct vitality were assessed macroscopically, before and after free transplantation, on a daily basis over an observation period of two weeks. Neovascularization was evaluated histologically and using selective microangiography. Formation of newly synthesized cartilage ECM was evaluated immunohistochemically.

#### Selective Microangiography and quantification of neovascularization

In order to carry out selective microangiography, animals were anaesthetised and i.v. heparinised (5.000 IU). The artery of the implanted vascular pedicle was cannulated with a catheter (0.9×25 cm) and the vascular system of the prefabricated skin flap was cleaned for 20 mins with heparinised saline solution (37°C). Furthermore, a suspension of Micropaque 30% and Rheomacrodex 10% in a ratio of 2∶1 was injected with a constant pressure of 110 mmHg, for 45 mins into the vascular system via the artery. Once the animals had been sacrificed using Narcoren 160 mg/kg (Merial, Hallbergmoos, Germany) the flaps were removed and pinned to cork mats using cannula in order to prepare them for microangiographic photography using a mammography device (Mammomat 3000 Nova, Siemens Healthcare AG, Erlangen, Germany). In the microangiographic control group 6 animals served to determine the existing pattern of vessels and the morphology in the abdominal skin flap using a Radifluor 120– TORR (Philips Electronic Instruments, LA, California). 25 evenly spaced standard integral lines were placed corresponding to the 15-cm flap length. Vessel quantity was determined by counting the total number of times that vessels perfused by Micropaque ® intersected the integral lines over the entire surface of the flap under 2× magnification as described previously [14]. The contrast of the microangiographic recordings of the 3D flaps was reduced using an image processing program (Photoshop CS3, Adobe, US), until the resolution of the small vessels matched that of the microangiographic control group.

#### Histology

After microangiographic photography, conventional histological samples were prepared. The samples were prepared with cuts orthogonal to the vascular pedicle. The staining was done using Hematoxylin Eosin (HE) Alcianblue (Ab) and Elastica van Gieson (EvG).

#### Immunohistochemistry

For immunohistochemical staining for the detection of collagen II Zytomed KIT (Broad Spectrum Kit, Zytomed, Berlin, Germany) was used according to manufacturer's instructions.

#### Statistical analysis

Two-tailed Mann-Whitney test was performed using GraphPad Prism (version 6.0b for Mac OS X, GraphPad Software, La Jolla, USA).

## Results

15 animals underwent surgery during this study. Three animals died around the time of implantation of the vascular pedicle. The remaining 12 flaps, with integrated auricular cartilage constructs could be successfully neovascularized. Four-sided flap excision was performed in every case and all skin flaps survived. After wound healing all flaps could be raised, and reanastomosed microsurgically to saphenous artery and greater saphenous vein. In two animals, however, the vascular pedicle tore on the second day following free transplantation and in one animal there was a thrombosis in the vascular pedicle and the flap was thus necrotic on the 8^th^ day post-operation and the animal was euthanized.

By means of an auricular cartilage biopsy, it was possible to obtain, on average, 170,260 cells following cell isolation. On average 3×10^5^ cells have been isolated per gram cartilage biopsy. Biomaterials of experimental and control group were seeded with 96 to 153.9 million cells following cell amplification. This corresponded, on average, to 28.5 million cells per cm^3^ of the biomaterial construct. It was possible to achieve an adequate cell number following a maximum of 3 passages. *Table 1* shows the cartilage and cellular data in monolayer culture.

**Table 1:** Harvesting of cartilage, cell culture, and seeding of biomaterials.

	weight of cartilage biopsy (g)	Number of cells after isolation	Number of cells after amplification
construct 1	0.63	8.1×10^4^	9.6×10^7^
construct 2	0.477	32.4×10^4^	9.6×10^7^
construct 3	0.33	25.8×10^4^	14.3×10^7^
construct 4	0.791	66.2×10^4^	15.4×10^7^
construct 5	0.45	39.9×10^4^	10.6×10^7^
construct 6	0.75	42.7×10^4^	11.1×10^7^
construct 7	0.538	3×10^4^	11.9×10^7^
construct 8	0.79	4.6×10^4^	13×10^7^
construct 9	0.79	4.2×10^4^	11.8×10^7^
construct 10	0.218	4.2×10^4^	9.7×10^7^
construct 11	0.37	5.5×10^4^	8.1×10^7^
construct 12	0.8	9.7×10^4^	13×10^7^
construct 13	0.701	9×10^4^	10.5×10^7^
construct 14	0.28	4.9×10^4^	9.7×10^7^
construct 15	0.48	6.5×10^4^	13×10^7^
control 1	0.67	16.1×10^4^	14×10^7^
control 2	0.51	6.7×10^4^	9.7×10^7^
**average**	**0.56**	**17.0**×**10^4^**	**11.5**×**10^7^**

The auricular cartilage biopsies were weighed prior to cell isolation after thorough cleaning and sectioning. After cell isolation cell number was determined. After amplification cell number was increased 676× after passage two or three. There were no significant differences between test and control group; p = 0.881 (weight of cartilage biopsy), p = 0.766 (cells isolated), p = 0.655 (amplificated cell number); Mann-Whitney Test (two-tailed).

### Cell Shape and vitality during in Vitro Culture

During amplification chondrocytes exhibited the typical pattern of dedifferentiation. Following the inoculation of chondrocytes in the biomaterial construct and further cultivation for one week, the cells underwent redifferentiation regaining an almost round, cartilage-specific shape. Using live/dead staining, vitality of almost 100% could be determined consistently *(* *fig. 1C* *)*.

### Flap Viability

In all experimental animals and in the control group, the post-operative appearance of the flap was inconspicuous. The growth of hair was normal throughout the entire observation period. The edges of the wounds consistently healed uneventful *(* *fig. 2C* *).* However, in some animals there was evidence of small, local infections at the edges of the wounds one to two weeks after the operation measuring a maximally 1.5×3.5 cm. Most likely they were caused by manipulation of the animals, scratching and consecutive wound-edge infection. This always occurred in the areas at the edge of the wounds and had no effect whatsoever on the overall vitality of the flap. In five of the ten freely transplanted skin flaps with integrated cultivated cartilage tissue there was evidence of venous congestion, beginning from the first day following the operation. This had generally abated by the second or third day after the operation at the latest. There were no signs of necrosis.

### Shape and Size of Tissue Engineered Auricles Remain Largely Unaltered

The cartilage constructs were integrated well into the flaps. Directly after implantation, the auricle was clearly and distinctly evident beneath the skin. The structures of the seeded cell carrier were clearly visible and palpable and were only covered by a thin layer of skin. The formation of a capsule around the auricle led to a more plump appearance of the initially visible filigree structures of the helix, antihelix and the tragus. Even after free transplantation, the implanted large auricles (13 pieces) were stable in terms of structure, palpable in the skin flap and remained well integrated. The two small implanted auricles, however, had a considerably altered form due to a lack of stability. Changes in form caused by the degradation of the biomaterial construct were not observed during the observation period of a maximum of 12 months. The cultivated auricular cartilage biomaterial constructs were of firm consistency similar to that of native cartilage. The control constructs were as well integrated into the flaps, and over time the formation of a capsule around the auricle led to a plump appearance. In contrast to the constructs with prefabricated skin flap they revealed reduced mechanical stability.

In four animals a seroma (25–50 ml) formed around the implanted construct after implantation. Bacteriological tests showed no evidence of germs.

### Selective Microangiography Demonstrates Neovascularization in the Complete Flap Area

A selective microangiography was carried out on all experimental animals after free transplantation and healing of incisions using a Mammomat 3000 Nova (Siemens Healthcare AG, Erlangen, Germany). Ten skin flaps were analysed. Two necrotic flaps could not be evaluated.

**Figure 3:**
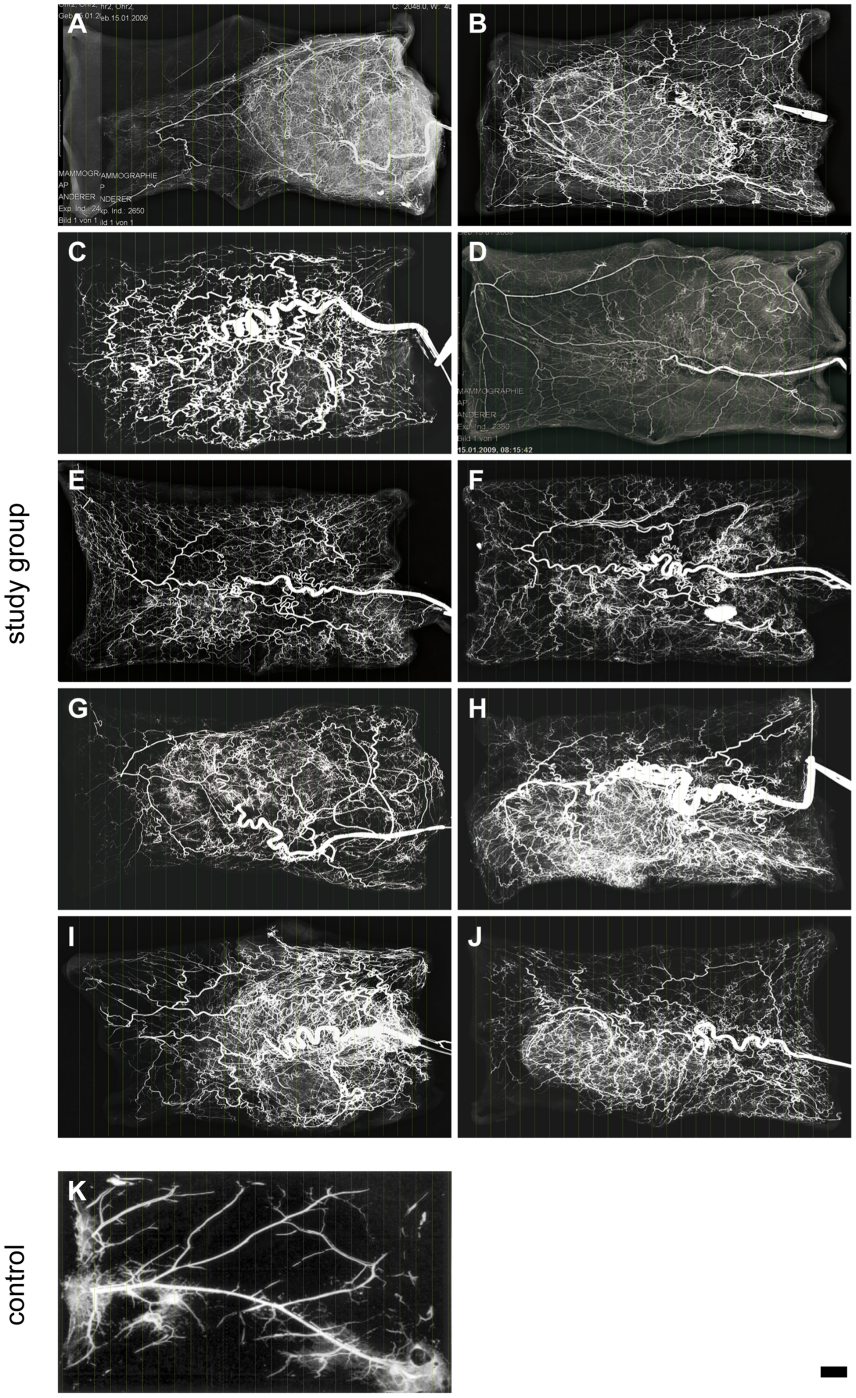
Microangiographic images of prefabricated 3D skinflaps after neovascularisation. A–J: Images show vasculature of abdominal skin flaps, with integrated 3D cartilage constructs, perfused by Micropaque ® through the implanted vascular pedicle. K: The micro-angiographic control was performed using a Radifluor 120– TORR (Philips Electronic Instruments, Los Angeles, USA). Scale bars = 1 cm.

**Figure 4:**
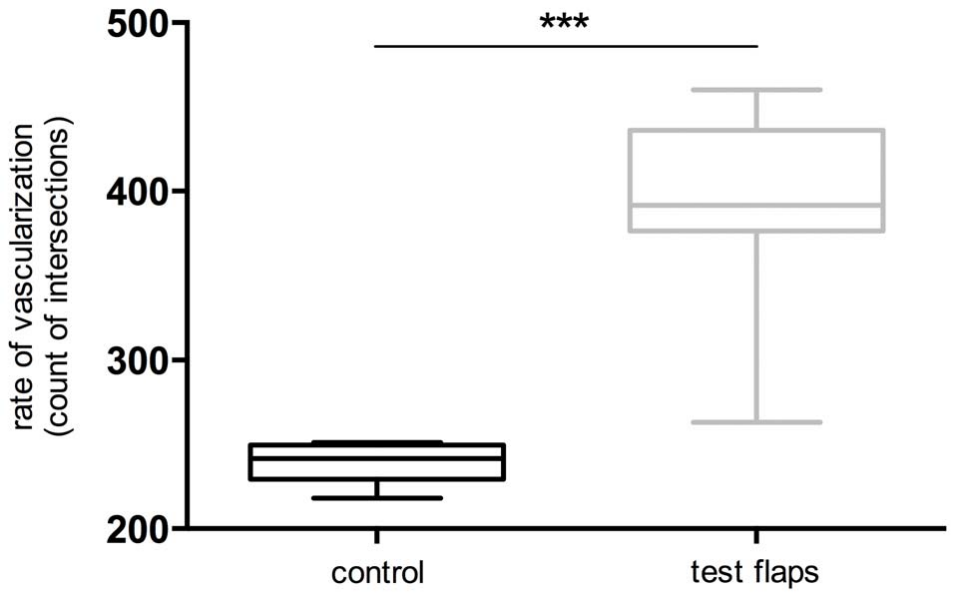
Angiographically quantified neovascularization in prefabricated 3D flaps. This boxplot shows the quantitative neovascularization of the 10 test flaps in comparison to 6 native abdominal skin flaps (control). To quantify the neovascularization in prefabricated 3D flaps 25 evenly spaced standard integral lines were placed corresponding to the 15-cm flap length. Vessel quantity was determined by counting the total number of times that vessels perfused by Micropaque ® intersected the integral lines over the entire surface of the flap under 2× magnification as described previously [14]. There was a significant difference between experimental and control group, ***p = 0.0002, Mann-Whitney Test (two-tailed).

The prefabricated, 3D skin flaps were all very well neovascularized. Starting with the vascular pedicle the vascular system showed complete coverage of all flaps, with the exception of one flap. In this animal, the vascular pedicle was torn. Thus the distal section of the flap was not completely filled with contrast agent – however, still this was shown macroscopically to be vital after the operation. In the area of the integrated auricular cartilage biomaterial construct the main area of neovascularization was evident through a fine and very thick network of vessels *(* *fig. 3* *)*. Vessel density in the flap had increased on average by 158% (range 110–193%) as a result of vascular pedicle implantation. The boxplots in *figure 4* illustrate the acquired quantitative values for the vessel count in prefabricated, 3D skin flaps with integrated, tissue engineered auricular cartilage in comparison to the control group.

### Histology

**Figure 5:**
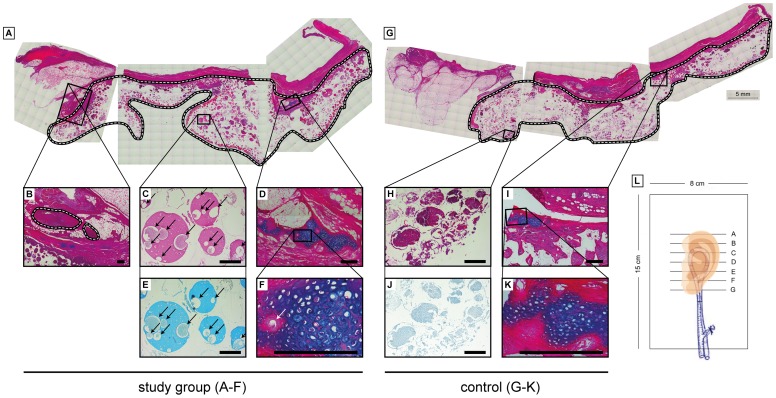
Histological results. A (study group): Stitched full cross section of the cultured auricle (black+white line). B: Implanted vascular pedicle (saphenous artery and greater saphenous vein), original magnification, x5. C+E: Low power view of the center of the cultivated cartilage cell biomaterial construct shows GAG, black arrows indicate ingrown vessels (C: Alcian blue counterstained with Kernechtred, original magnification, x50; E: Alcian blue staining, original magnification, x50). D+F: Periphery of cultivated cartilage cell biomaterial construct shows vital cartilage and good integration in the skin flap. White arrow indicates an ingrown vessel (Alcian blue counterstained with Kernechtred, original magnification, D×10; C×200). G (control group): Stitched full cross section of the cultured auricle (black+white line). H+J: Low power view of the center of the cultivated cartilage cell biomaterial construct shows necrosis with no GAG (H: Alcian blue counterstained with Kernechtred, original magnification, ×50; J: Alcian blue staining, original magnification, ×50). I+K: Periphery of cultivated cartilage cell biomaterial construct shows small areas of vital cartilage and good integration in the skin flap. (Alcian blue counterstained with Kernechtred, original magnification, I×10, K×200). L: Image indicates the performed sectional planes within the biomaterial construct. All showed histological images are from section plane E. Scale bars = 400 µm.

The skin was intact in all specimens. The implanted vascular pedicles exhibited a regular morphological pattern. Around the cartilage constructs there was histological proof of good neovascularization in all specimens *(* *fig. 5C, E, F* *)*.

In the area of the implanted, tissue engineered cell constructs, cartilage-like cells were found on their own and grouped as part of small cell structures. Large vital areas with cartilage-like tissue containing a significant amount of collagen II, as shown both in the alcian staining and immunohistochemically, were found mostly in the areas at the periphery of the construct *(* *fig. 5D, F* *)*. Some central areas also contained cartilage-like cells and collagen II *(* *fig. 5C, E* *)*. However, these were not as redifferentiated as the areas located at the periphery of the constructs. The central cells were arranged in a more sparse fashion and were surrounded by many fibres and an extra-cellular matrix. In the control group only small vital areas with cartilage-like tissue were found at the periphery of the construct *(* *fig. 5I, K* *)*. The center of the scaffold showed no vital cells, but instead large areas of necrosis *(* *fig. 5H, J* *)*.

The biomaterial construct did not fully degrade during observation periods ranging between 9 and 11 months after implantation in any of the animals, either in the study group nor in the control group. Degradation took place mainly at the periphery of the constructs. Centrally, the construct material appeared almost unchanged. In the area of the biomaterial (both in the study and the control group) there was evidence, in some of the specimens, of accumulations of leucocytes.

## Discussion

The ultimate objective of the study described here is the transplantation of neovascularized, cultivated and formed cartilage tissue for the 3D reconstruction of complex cartilage defects. The vascularization of large, 3D cultivated tissue volumes was realized within this work by means of the prefabrication of flaps using an autologous model. These vital, manufactured connective tissue flaps could, following sufficient neovascularization in the skin flaps and around the bioconstructs at the recipient location, be transplanted to the defective cartilage area through free transplantation. This also offers the possibility of implanting the cultivated tissue in suitable locations, which fulfill both the aesthetic and functional requirements. For a particularly demanding tissue with regard to sufficient circulation, this represents a technology for the neovascularization of the biomaterial construct prior to cell seeding.

If an engineered biomaterial construct is implanted into a prefabricated flap, in this way a neovascularized, defect-specific, 3D tissue flap can be created to meet individual requirements. In addition, it appears feasible to use the neovascularization of the biomaterial construct separately, in a targeted manner. It would be possible to neovascularize all of the tissue cultivated using the tissue engineering procedure.

In recent literature, various additional approaches to improving the supply of nutrients in cultivated cell constructs have been described. This includes the use of growth factors, bioactive materials, cell transplants, targeted cell cultivation and the manipulation of physiological neoangiogenesis under hypoxic conditions [15]. Cell carriers from the field of nanotechnology with already-integrated channels for the improvement of diffusion also produced positive results [16]. Overall, these approaches show some success, but are, mostly quite expensive and cannot be applied to all types of tissue.

A significant number of authors have reported that the deposition of the extracellular matrix takes place mainly at the periphery of the engineered cartilage. In contrast, the central region of the cultivated tissue contains fewer cells and matrix [2], [4]. According to Matin this phenomenon can be traced back to the uneven cell seeding within the construct pores and to diffusion limitations in the internal area [17]. In our study an uneven cell seeding can be excluded, as many vital chondrocytes have been found in the center of the construct after seeding. Cultivated 3D chondrocyte constructs within neovascularized skin flap produced cartilage-specific extracellular matrix (ECM) components, such as GAGs and collagen even in the center of the constructs.

Experimental in-vivo tests regarding tissue engineering of cartilage have previously been conducted mostly in nude mice, which have a compromised immune system [18], [19]. In order to lead experimental work closer to clinical application, autologous cartilage tissue was used in this immunocompetent animal study. In addition, because no immunosuppression was used, it was possible to evaluate the immunological potential of the chondrocyte cell biomaterial constructs.

In four animals, which were operated on at an early stage during the study, there was clear evidence of seroma formation around the implanted construct following construct implantation. We hypothesize that a component of the medium – most likely foetal calf serum – was responsible for this allergic/inflammatory reaction. Once the auricular cartilage construct had been rinsed with in saline prior to implantation, no more seroma were. A slight swelling and reddening was, however, observed in most of the animals, which abated after a few days. It can therefore be assumed that the foetal calf serum indeed contributed considerably to the immunological reaction, but was not solely responsible [17], [20]. Other studies confirm the immunological potential of foetal calf serum. It is therefore recommendable to use serum-free culture media [21], [22] or carry out sufficient cleaning before implantation. Alternatively, autologous serum might be suitable [21]. The potential of non-human serum for infection with prion diseases should be acknowledged. Moreover, the implanted silicon foil can be responsible for foreign body reactions [2], [23]–[25].

The selective microangiography demonstrated that skin flaps with integrated cultivated cartilage cell constructs were successfully neovascularized. In particular, the area of the biomaterial construct was rich in fine vessels. The quantitative analysis, viewed in comparison with the microangiographic control group (the native existing anatomical vessel density in 6 animals), showed that the vessel density in the flap had increased by 110–193% as a result of the vascular pedicle implantation. There was a significant difference between experimental and control group, p = 0.0002, Wilcoxon-Mann-Whitney Test. The increase of vessels, achieved by flap prefabrication, leads to a better vascular integration and thus better supply of nutrients and oxygen in cultured cartilage. If one were to compare these values with the neovascularization in the flaps without implanted constructs, as described in the available literature [7], these values are somewhat higher. This indicates that the implanted scaffold has a positive influence on the neovascularization. There were numerous vessels found histologically, within a fibrous capsule on the surface of the implanted cartilage constructs. In contrast, in the area of cultured cartilage, there was no neovascularization found. This was most likely due to inhibitory components of cultured cartilage like troponin I as described by Moses et. al. [26]. Thus prefabrication, by means of the implantation of a vascular loop, improves vascular integration without negative influence or dedifferentiation of implanted cartilage.

In the control group without implanted vascular pedicle, only small vital areas with cartilage-like tissue were found at the periphery of the construct. The center of the scaffold did not contain vital cells, but instead large areas of necrosis. In the study group some central areas also contained cartilage-like cells and collagen II, but in none of the constructs cartilage-like tissue was found throughout. In order to resolve this problem, an improved nutrient supply to the centre of the construct, among other solutions, will be required, which has so far been limited by the existing diffusion limits in the construct [17].

### Conclusions

Adopting an autologous model it was possible, to realise a combination of prefabricated skin flaps by means of vascular pedicle implantation and the implantation of *in vitro* cultivated chondrocytes bioconstructs for the manufacture of functional, 3D cartilage grafts.

In this study, cartilage biopsies were taken from the auricles of 15 chinchilla-bastard rabbits, multiplied in monolayer culture and then seeded in a 3D cell carrier made from polycaprolactone. The chondrocyte biomaterial constructs were implanted in the skin flaps and then neovascularized using vascular loops with terminal arteriovenous anastomosis. They were then grown as connective tissue flaps and reimplanted in the same position, microsurgically.

It was possible to show macroscopically, that the combined, prefabricated 3D flaps could be freely transplanted. The integrated 3D constructs were stably integrated within the skin flaps. It was shown microangiographically that all skin flaps with implanted, cultivated constructs were well neovascularized. There was evidence of cartilage-like tissue with the formation of cartilaginous ECM even in the center of large cultured cartilage constructs. It was possible to demonstrate the presence of newly formed collagen type II. In contrast, in the control group, without implanted vascular loop, only small vital areas with cartilage-like tissue were found at the periphery of the construct. The center of the scaffold did not contain vital cells, but instead large areas of necrosis. Results of the experiment demonstrated that cultivated 3D auricular cartilage constructs with prefabricated skin flaps could be combined by means of vascular pedicle implantation and could be transplanted. With respect to future clinical application, the procedure is a promising alternative for clinical practice because of favorable esthetic outcomes with minimal donor site morbidity.
