# Correction: Prefabrication of 3D Cartilage Contructs: Towards a Tissue Engineered Auricle – A Model Tested in Rabbits

**DOI:** 10.1371/journal.pone.0113017

**Published:** 2014-11-03

**Authors:** The PLOS ONE Staff

The last two authors, Katharina Storck and Hoang Nguyen The, should be noted as contributing equally to this work.
